# Comparison of bleeding complications after pediatric kidney biopsy between intravenous sedation and general anesthesia: a nationwide cohort study

**DOI:** 10.1186/s12887-022-03828-8

**Published:** 2023-01-20

**Authors:** Akira Okada, Kayo Ikeda Kurakawa, Yutaka Harita, Akira Shimizu, Satoko Yamaguchi, Shotaro Aso, Sachiko Ono, Yohei Hashimoto, Ryosuke Kumazawa, Nobuaki Michihata, Taisuke Jo, Hiroki Matsui, Kiyohide Fushimi, Masaomi Nangaku, Toshimasa Yamauchi, Hideo Yasunaga, Takashi Kadowaki

**Affiliations:** 1 Department of Prevention of Diabetes and Lifestyle-Related Diseases, Graduate School of Medicine, the University of Tokyo, Tokyo, Japan; 2 Department of Pediatrics, National Rehabilitation Center for Persons with Disabilities, Namiki, Saitama Japan; 3 Department of Pediatrics, Graduate School of Medicine, the University of Tokyo, Bunkyo-ku, Tokyo, Japan; 4 Department of Analytic Human Pathology, Graduate School of Medicine, Nippon Medical School, Tokyo, Japan; 5 Department of Biostatistics and Bioinformatics, Graduate School of Medicine, the University of Tokyo, Tokyo, Japan; 6 Department of Eat-Loss Medicine, Graduate School of Medicine, the University of Tokyo, Tokyo, Japan; 7 Department of Clinical Epidemiology and Health Economics, the University of Tokyo, Tokyo, Japan; 8 Department of Ophthalmology, Graduate School of Medicine, the University of Tokyo, Tokyo, Japan; 9 Center for Clinical Sciences, National Center for Global Health and Medicine, Tokyo, Japan; 10 Department of Health Services Research, Graduate School of Medicine, the University of Tokyo, Tokyo, Japan; 11 Department of Respiratory Medicine, Graduate School of Medicine, the University of Tokyo, Tokyo, Japan; 12 Department of Health Policy and Informatics, Tokyo Medical and Dental University, Tokyo, Japan; 13 Division of Nephrology and Endocrinology, Graduate School of Medicine, the University of Tokyo, Tokyo, Japan; 14 Department of Diabetes and Metabolism, Graduate School of Medicine, the University of Tokyo, Tokyo, Japan; 15 Toranomon Hospital, 2-2-2 Toranomon, Minato-ku, 105-8470 Tokyo, Japan

**Keywords:** Pediatric kidney biopsy, Anesthesia, Kidney disease, Bleeding complications, Clinical epidemiology

## Abstract

**Background:**

An increasing number of studies are evaluating the safety of intravenous sedation compared with that of general anesthesia; however, data on bleeding complications after pediatric percutaneous renal biopsy performed under intravenous sedation or general anesthesia are lacking. We aimed to examine differences in bleeding complications between intravenous sedation and general anesthesia in pediatric patients.

**Methods:**

Data of pediatric patients aged ≤ 15 years undergoing percutaneous kidney biopsy for kidney disease between July 2007 and March 2019 were retrieved from a national inpatient database in Japan. We examined differences in bleeding complications after renal biopsy performed under intravenous sedation, defined by the absence of the record of general anesthesia with intubation but by the presence of intravenous sedation during biopsy, and general anesthesia, defined by the presence of the record of general anesthesia with intubation during biopsy, among pediatric patients admitted for percutaneous renal biopsy. We performed binomial regression using overlap weights based on propensity scores for patients receiving intravenous sedation. Analyses stratified by age or sex, a sensitivity analysis using generalized estimating equations considering cluster effects by hospital among a propensity score-matched cohort, and another sensitivity analysis using the instrumental variable method were performed to confirm the robustness of the results.

**Results:**

We identified 6,560 biopsies performed in 5,999 children aged 1–15 years from 328 hospitals and 178 events. Only three severe complications and no death were observed. No significant difference in the proportion of bleeding complications was observed between procedures performed under intravenous sedation and those performed under general anesthesia (unadjusted proportions, 2.8% and 2.3%; adjusted proportions, 2.5% and 2.2%), with an unadjusted relative risk of 1.21 (95% confidence interval, 0.80–1.81) and adjusted relative risk of 1.13 (95% confidence interval, 0.74–1.73). Both age- and sex-stratified analyses yielded similar results. The analysis using generalized estimating equation and the instrumental variable method showed relative risks of 0.95 (95% confidence interval, 0.48–1.88) and 1.18 (95% confidence interval, 0.74–1.89), respectively.

**Conclusion:**

This retrospective cohort study using a national database revealed that the risk of biopsy-related bleeding was comparable between intravenous sedation and general anesthesia during pediatric percutaneous kidney biopsy, suggesting that intravenous sedation alone and general anesthesia may have a similar bleeding risk in pediatric percutaneous kidney biopsies.

## Background

Kidney disease places a burden not only on healthcare services, but also on the economic and social lives of patients [1], and therefore, detection of its etiology is important, especially in pediatric patients. Kidney biopsy is the gold standard diagnostic tool to elucidate the pathophysiology of kidney disease [2]. However, although kidney biopsy is of pivotal importance in pediatric nephrology, nephrologists pay attention to its invasiveness and try to reduce the occurrence of complications, including hematuria, retroperitoneal hematoma, arteriovenous fistula, and uncontrollable bleeding that ultimately leads to death [3].

Although there are a number of reports regarding complications following kidney biopsy in adult patients, fewer reports are available on complications after pediatric kidney biopsy. For example, a meta-analysis including 118,064 biopsies performed in adult patients is available [3], while the largest meta-analysis including pediatric patients involves 5,504 biopsies [4]. Since complication rates following kidney biopsy are drastically different between pediatric and adult patients [3, 4], studies are warranted to elucidate details regarding complications following pediatric kidney biopsy.

General anesthesia or intravenous sedation has been traditionally used in young children undergoing invasive procedures or painful or anxiety-provoking procedures. These procedures are also used when immobilization of patients is required, such as during cardiac catheterization [5–7]. There are an increasing number of studies evaluating the safety and effectiveness of intravenous sedation in comparison with that of general anesthesia [5, 6]. However, no previous studies have compared bleeding complications after pediatric percutaneous kidney biopsies performed under intravenous sedation or general anesthesia. Even a meta-analysis of complications following pediatric kidney biopsies failed to evaluate the risk of anesthesia or sedation [4]. In this study, we aimed to examine the difference in bleeding complications after kidney biopsy between procedures performed under intravenous sedation and those performed under general anesthesia in pediatric patients using a national inpatient database in Japan.

## Methods

### Data source

The Japanese Diagnosis Procedure Combination database is a nationwide administrative database of claims and discharge abstract data that has been previously described in detail [8]. The database includes data for approximately 7 million inpatients per year from more than 1000 participating hospitals and covers approximately 90% of all tertiary-care hospitals in Japan. A previous validation study confirmed that the recorded procedures had high sensitivity and specificity [9]. The database includes the following information: age, sex, body mass index (BMI), diagnosis recorded using the International Classification of Diseases, 10th revision (ICD-10) codes and free-text data in Japanese, level of consciousness at admission, and discharge status. Additionally, the database includes data on procedures performed and drugs used during hospitalization [8].

This study was conducted in accordance with the Helsinki Declaration of 1975, as revised in 2013, and approved by the institutional review board of The University of Tokyo (approval no. 2018030NI). The need for informed consent was waived due to the anonymity of the data.

### Study design and population

Using the database, we identified patients aged 1–15 years who underwent percutaneous kidney biopsy under intravenous sedation or general anesthesia between July 1, 2010 and March 31, 2019. We defined the use of intravenous sedation only by the absence of the record of general anesthesia with intubation use but by the presence of intravenous sedation use during biopsy; we defined the use of general anesthesia by the presence of the record of general anesthesia with intubation use during biopsy. Patients scheduled for admission with a definite or suspected diagnosis of kidney disease (ICD-10 codes: D690, N00-08, N11, N12, N14, N158, N159, N16, N17, N18, N19, N25, N289, N391, N392, R31, or R80) who underwent a kidney biopsy within 4 days after admission were included. We excluded patients with impaired consciousness at admission; those who received kidney replacement therapy, mechanical ventilation, vasopressors, or a surgical procedure under general anesthesia before the kidney biopsy; those admitted to the intensive care unit; or those with a transplanted kidney (ICD-10 codes: Z940 or T861) or malignancy (C or D0). We excluded patients with transplanted kidney because the location of the transplanted kidney may differ from that of the naive kidney, and therefore the risk of bleeding at the time of biopsy can vary according to a previous study. [10] Patients who received blood transfusion or invasive hemostasis before kidney biopsy and those with missing BMI data were also excluded.

### Study variables

We extracted data on comorbidities at admission, including metabolic disease (ICD-10 codes starting with E), mental disease (F), neurological disease (G), cardiovascular disease (I), respiratory disease (J), musculoskeletal disease (M), and congenital disease (Q). We also extracted data on the etiology of kidney disease recorded in the database (chronic nephritis or nephrotic syndrome), use of corticosteroids or immunosuppressants before kidney biopsy, use of albumin infusion before kidney biopsy, use of tranexamic acid on the day of kidney biopsy (because a randomized controlled trial showed its effect on reducing bleeding events [11]), type of anesthesia used during kidney biopsy (general anesthesia or intravenous sedation only), and history of previous kidney biopsy during the study period. We also obtained information on hospital characteristics and the year when the biopsy was performed because these factors may be associated with physician preference for application of a certain sedation method for biopsy, and possibly complication occurrence [12, 13]. We defined the hospital volume for pediatric kidney biopsies as the average annual number of pediatric patients who underwent percutaneous kidney biopsy and made a binary variable indicating whether the hospital was an academic hospital.

Age was categorized into three groups: 1–5, 6–11, and 12–15 years. BMI was categorized based on the BMI standard deviation score (BMI-SDS) for Japanese children [14]: underweight (BMI-SDS of < -1.28), normal weight (BMI-SDS of -1.28 to 1.279), and overweight (BMI-SDS of ≥ 1.28). Hospital volume was categorized as low, medium, or high based on the rounded tertiles of the included hospitals.

### Outcomes

The primary outcome was the occurrence of bleeding complications. Severe bleeding complications were defined as either red cell transfusion within 7 days after kidney biopsy or invasive hemostasis (transcatheter arterial embolization or nephrectomy). Non-severe complications were defined as diseases occurring after hospitalization according to ICD-10 codes denoting hemorrhagic events or hematoma formation, such as acute hemorrhagic anemia. The codes included hemorrhagic anemia (D500), retroperitoneal hemorrhage (K661), retroperitoneal hematoma (K668), renal or perirenal hemorrhage (N288), hemorrhagic shock (R571), hemorrhage occurring following the procedure (R58), renal hematoma (S3700), perirenal hematoma (T140), post-procedure hemorrhage or hematoma (T810), and hemorrhagic shock (T794), as defined in a previous study examining post-biopsy complications [13].

### Statistical analysis

We summarized the patient characteristics in the intravenous sedation and general anesthesia groups. Patient characteristics were compared using Fisher’s exact test for categorical variables and Student’s t-test for continuous variables. We also described the length of hospital stay and total hospitalization costs for each group. The total hospitalization costs were converted into US dollars ($), assuming that 120 Japanese yen was equivalent to $1.

We used propensity scores to minimize confounding caused by indications. When we estimated the propensity scores for receiving intravenous sedation only, we used a logistic regression model, setting the dependent variable as the receipt of intravenous sedation and the independent variables as all the above-listed covariates. Based on the calculated propensity scores, we conducted an analysis using overlap weights to adjust for the differences [15]. Overlap weights were defined as 1 − propensity score among patients receiving intravenous sedation only and as the propensity score among patients receiving general anesthesia. Thereafter, we used weighted generalized linear models with binomial distribution and log link functions to obtain the relative risks. To calculate the relative risks with overlap weights in both groups, we used robust variance estimators to calculate confidence intervals (CIs) as used in weighted analyses [16]. We also calculated risk differences in the primary analysis using a generalized linear model with a Gaussian distribution, identity link function, and robust variance.

#### Stratified analysis

We performed analyses stratified by age and sex with and without overlap weights. In the age-stratified analyses, we used the age in months to adjust for individual age.

#### Sensitivity analysis

Two sensitivity analyses of the outcomes were performed. First, we performed propensity score matching instead of overlapping weights. Using this propensity score-matched cohort, we used generalized estimating equations with binomial outcome distribution, log link function, exchangeable working correlation model, and sandwich variance estimator with each hospital set as a unit of a cluster; this method can adjust for the effects of hospital clustering [17, 18]. Second, we used the instrumental variable method to confirm causal inference. Instrumental variable analysis can theoretically adjust for both measured and unmeasured confounders between two groups [19, 20]. Each hospital’s preference for intravenous sedation only was selected as an instrumental variable because the choice of intravenous sedation presumably depends only on physician preference [19]. We used two-stage residual inclusion estimation for the instrumental variable analysis [21, 22].

We used a two-sided significance level of 0.05 and performed all statistical analyses using Stata version 17 (StataCorp, College Station, TX, USA).

## Results

**Fig. 1:**
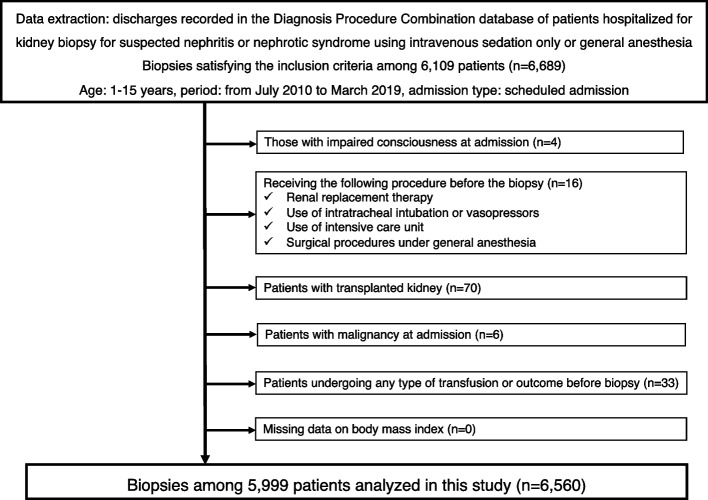
Flow chart of the patient selection process

Among the 6,689 percutaneous kidney biopsies that met the inclusion criteria, 129 were excluded (Fig. 1). The remaining 6,560 biopsies were performed in 5,999 patients at 328 hospitals, and 178 biopsies reached the outcome. Of the 178 biopsies, three patients underwent transfusion or invasive hemostasis procedures, while the remaining 174 patients experienced hemorrhagic events or hematoma formation. Severe complications occurred only in one patient under general anesthesia (1/1,164, 0.09%) and two patients under intravenous sedation (2/5,396, 0.04%). Of these three patients, two underwent red blood cell transfusion and two received transcatheter arterial embolization (due to overlapping); none experienced surgical nephrectomy. Thus, most of the bleeding events detected after percutaneous kidney biopsy were not severe. None of the included patients died during hospitalization.

The background characteristics of the included patients are presented in Table 1. Patients from the intravenous sedation group were more likely to be older and to have chronic nephritis, low BMI, to be hospitalized in academic hospitals, and to have a biopsy history at the same hospital. The mean length of stay and total hospitalization costs were longer and higher, respectively, in patients undergoing general anesthesia. The outcome occurred in 2.3% (27 out of 1164 biopsies) of children receiving general anesthesia and in 2.8% (151 out of 5396 biopsies) of children receiving intravenous sedation. The unadjusted relative risk and risk difference (vs. general anesthesia) were 1.21 (95% CI: 0.80–1.81) and 0.48% (95% CI: -0.48–1.44%), respectively.

**Table 1:** **Table 1** Characteristics of eligible children categorized by anesthesia method

Variable	Category	Using general anesthesia	Using intravenous sedation only	*P*-value
		*n*=1164	*n*=5396	
Age	1–5 years	358 (30.8%)	876 (16.2%)	<0.001
	6–11 years	619 (53.2%)	2568 (47.6%)	
	12–15 years	187 (16.1%)	1952 (36.2%)	
Male		682 (58.6%)	3035 (56.2%)	0.14
Body mass index	Underweight	93 (8.0%)	536 (9.9%)	0.007
	Normal	859 (73.8%)	4046 (75.0%)	
	Overweight	212 (18.2%)	814 (15.1%)	
Kidney disease	Nephritis	725 (62.3%)	3519 (65.2%)	0.058
	Nephrotic syndrome	439 (37.7%)	1877 (34.8%)	
Comorbid kidney failure		9 (0.8%)	36 (0.7%)	0.69
Acute kidney injury/acute or rapidly progressive disease		16 (1.4%)	81 (1.5%)	0.75
Use of antiplatelets or anticoagulants		11 (0.9%)	104 (1.9%)	0.021
Comorbid anemia on admission		18 (1.5%)	38 (0.7%)	0.005
Coagulation disorders on admission		11 (0.9%)	37 (0.7%)	0.35
Metabolic disease		70 (6.0%)	311 (5.8%)	0.74
Mental disease		18 (1.5%)	58 (1.1%)	0.17
Neurological disease		15 (1.3%)	38 (0.7%)	0.043
Cardiovascular disease		215 (18.5%)	797 (14.8%)	0.002
Respiratory disease		137 (11.8%)	470 (8.7%)	0.001
Musculoskeletal disease		67 (5.8%)	376 (7.0%)	0.14
Congenital disease		12 (1.0%)	40 (0.7%)	0.31
Use of corticosteroids		137 (11.8%)	565 (10.5%)	0.19
Use of immunosuppressants		142 (12.2%)	679 (12.6%)	0.72
Use of tranexamic acid		529 (45.4%)	2189 (40.6%)	0.002
Use of albumin infusion		9 (0.8%)	47 (0.9%)	0.74
Hospital volume (cases/year)	1–5	491 (42.2%)	1628 (30.2%)	<0.001
	6–12	396 (34.0%)	1804 (33.4%)	
	13–	277 (23.8%)	1964 (36.4%)	
Academic hospital admission		486 (41.8%)	2394 (44.4%)	0.10
History of biopsy at the same hospital		136 (11.7%)	743 (13.8%)	0.058
Fiscal year	2010	59 (5.1%)	397 (7.4%)	<0.001
	2011	73 (6.3%)	468 (8.7%)	
	2012	129 (11.1%)	631 (11.7%)	
	2013	141 (12.1%)	694 (12.9%)	
	2014	173 (14.9%)	790 (14.6%)	
	2015	171 (14.7%)	821 (15.2%)	
	2016	190 (16.3%)	797 (14.8%)	
	2017	228 (19.6%)	798 (14.8%)	
Length of stay (days)		11.0 (14.3)	9.0 (11.2)	<0.001
Total hospitalization costs (US dollars)		4986 (5531)	3381 (4648)	<0.001
Complication following biopsy		27 (2.3%)	151 (2.8%)	0.43

Data are presented as mean (standard deviation) for continuous measures and as n (%) for categorical measures

**Fig. 2:**
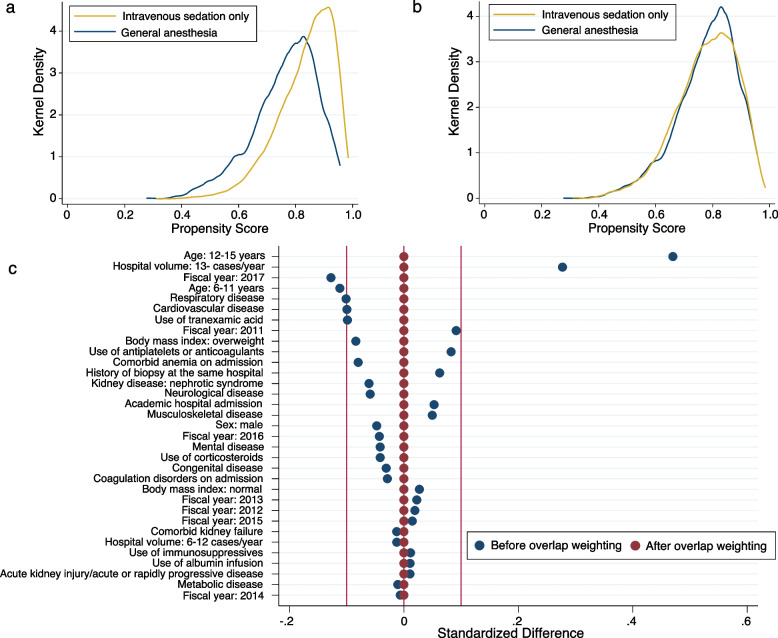
Distributions of propensity scores. Propensity scores from patients who received general anesthesia and intravenous sedation only (**a**) before and (**b**) after overlap weighting. **c** The standardized differences before and after overlap weighting in descending order of the absolute standardized differences before weighting

We calculated propensity scores to adjust for the receipt of intravenous sedation only, and the c-statistic of the propensity scores was 0.69. The calculated odds ratios for receipt of intravenous sedation only are shown in Table 2. As age increased, the possibility of receiving intravenous sedation only increased, while children with cardiovascular comorbidities were less likely to receive intravenous sedation only. Increased hospital volumes and recent fiscal years were also associated with the increased probability of receiving intravenous sedation only. After using overlap weights based on propensity scores, the distributions of the patient and hospital characteristics were well balanced (Table 3; Fig. 2c). The distributions of the propensity scores in the unweighted and weighted models are shown in Fig. 2a and b.

**Table 2:** **Table 2** Coefficients used in calculating propensity scores for the receipt of intravenous sedation only during percutaneous kidney biopsy

Variable	Category	Odds ratio	95% confidence interval			*p*-value
Age	1−5 years	Reference				
	6−11 years	1.81	1.54	–	2.12	<0.001
	12−15 years	4.97	4.04	–	6.12	<0.001
Sex	Female	Reference				
	Male	0.95	0.83	–	1.09	0.45
Body mass index	Underweight	Reference				
	Normal	0.97	0.76	–	1.24	0.80
	Overweight	0.88	0.66	–	1.17	0.38
Kidney disease category	Nephritis	Reference				
	Nephrotic syndrome	1.02	0.87	–	1.19	0.83
Comorbid kidney failure		0.86	0.40	–	1.86	0.70
Acute kidney injury/acute or rapidly progressive disease		1.14	0.64	–	2.02	0.66
Comorbid anemia on admission		0.50	0.27	–	0.93	0.027
Coagulation disorders on admission		0.83	0.41	–	1.70	0.62
Use of antiplatelet or anticoagulants		1.85	0.97	–	3.52	0.06
Metabolic disease		1.00	0.75	–	1.34	0.99
Mental disease		0.84	0.47	–	1.50	0.56
Neurological disease		0.54	0.28	–	1.03	0.06
Cardiovascular disease		0.73	0.61	–	0.87	0.001
Respiratory disease		0.84	0.68	–	1.04	0.11
Musculoskeletal disease		1.20	0.90	–	1.60	0.22
Congenital disease		0.74	0.37	–	1.48	0.40
Use of corticosteroids		0.97	0.77	–	1.24	0.82
Use of immunosuppressants		0.97	0.76	–	1.23	0.80
Use of tranexamic acid		0.68	0.60	–	0.78	<0.001
Use of albumin infusion		1.63	0.77	–	3.46	0.20
Hospital volume (cases/year)	1-5	Reference				
	6-12	1.37	1.16	–	1.61	<0.001
	13-	2.61	2.20	–	3.10	<0.001
Academic hospital admission		1.27	1.09	–	1.46	0.002
History of biopsy at the same hospital		1.25	1.00	–	1.55	0.047
Fiscal year	2010	Reference				
	2011	0.95	0.65	–	1.39	0.80
	2012	0.68	0.48	–	0.96	0.030
	2013	0.65	0.46	–	0.91	0.013
	2014	0.57	0.41	–	0.80	0.001
	2015	0.64	0.46	–	0.90	0.009
	2016	0.55	0.40	–	0.77	<0.001
	2017	0.44	0.32	–	0.61	<0.001

**Table 3:** **Table 3** Characteristics of eligible patients before and after overlap weighting

		Before overlap weighting			After overlap weighting		
Variable	Category	General anesthesia	Using intravenous sedation only	ASD	General anesthesia	Using intravenous sedation only	ASD
Age	1–5 years	30.8%	16.2%	34.8%	26.7%	26.7%	0.0%
	6–11 years	53.2%	47.6%	11.2%	54.5%	54.5%	0.0%
	12–15 years	16.1%	36.2%	47.0%	18.8%	18.8%	0.0%
Sex	Male	59.0%	56.0%	4.7%	58.0%	58.0%	0.0%
Body mass index	Underweight	8.0%	9.9%	6.8%	8.3%	8.3%	0.0%
	Normal	73.8%	75.0%	2.7%	74.0%	74.0%	0.0%
	Overweight	18.2%	15.1%	8.4%	17.7%	17.7%	0.0%
Kidney disease	Nephritis	62.3%	65.2%	6.1%	63.0%	63.0%	0.0%
	Nephrotic syndrome	37.7%	34.8%	6.1%	37.0%	37.0%	0.0%
Comorbidity	Kidney failure	1.0%	1.0%	1.3%	1.0%	1.0%	0.0%
	Acute kidney injury/acute or rapidly progressive disease	1.0%	2.0%	1.1%	1.0%	1.0%	0.0%
	Comorbid anemia on admission	2.0%	1.0%	8.0%	1.0%	1.0%	0.0%
	Coagulation disorders on admission	1.0%	1.0%	2.9%	1.0%	1.0%	0.0%
	Metabolic disease	6.0%	6.0%	1.1%	6.0%	6.0%	0.0%
	Mental disease	2.0%	1.0%	4.1%	1.0%	1.0%	0.0%
	Neurological disease	1.0%	1.0%	5.9%	1.0%	1.0%	0.0%
	Cardiovascular disease	18.0%	15.0%	10.0%	17.0%	17.0%	0.0%
	Respiratory disease	12.0%	9.0%	10.1%	11.0%	11.0%	0.0%
	Musculoskeletal disease	6.0%	7.0%	5.0%	6.0%	6.0%	0.0%
	Congenital disease	1.0%	1.0%	3.1%	1.0%	1.0%	0.0%
Use of corticosteroids		12.0%	10.0%	4.1%	11.0%	11.0%	0.0%
Use of immunosuppressants		12.0%	13.0%	1.2%	12.0%	12.0%	0.0%
Use of antiplatelet or anticoagulants		1.0%	2.0%	8.3%	1.0%	1.0%	0.0%
Use of tranexamic acid		45.0%	41.0%	9.9%	44.0%	44.0%	0.0%
Use of albumin infusion		1.0%	1.0%	1.1%	1.0%	1.0%	0.0%
Hospital volume (cases/year)	1–5	42.2%	30.2%	25.2%	39.9%	39.9%	0.0%
	6–12	34.0%	33.4%	1.2%	34.4%	34.4%	0.0%
	13–	23.8%	36.4%	27.7%	25.8%	25.8%	0.0%
Academic hospital admission		42.0%	44.0%	5.3%	42.0%	42.0%	0.0%
History of biopsy at the same hospital		12.0%	14.0%	6.3%	12.0%	12.0%	0.0%
Fiscal year	2010	5.1%	7.4%	9.5%	5.3%	5.3%	0.0%
	2011	6.3%	8.7%	9.1%	6.5%	6.5%	0.0%
	2012	11.1%	11.7%	1.9%	11.2%	11.2%	0.0%
	2013	12.1%	12.9%	2.3%	12.1%	12.1%	0.0%
	2014	14.9%	14.6%	0.6%	15.0%	15.0%	0.0%
	2015	14.7%	15.2%	1.5%	14.8%	14.8%	0.0%
	2016	16.3%	14.8%	4.3%	16.3%	16.3%	0.0%
	2017	19.6%	14.8%	12.7%	18.8%	18.8%	0.0%

*ASD* absolute standardized difference

Table 4 presents the results of the regression analysis for the outcome. Patients who received intravenous sedation only had a comparable risk of bleeding complications, with an adjusted relative risk of 1.13 (95% CI: 0.74–1.73) and a risk difference of 0.29% (95% CI: -0.67–1.25%).

**Table 4:** **Table 4** Relative risks for bleeding complications involving intravenous sedation in comparison with general anesthesia in the main, stratified, and sensitivity analyses

Analysis type	Population	Model	Relative risk	95% confidence interval			*P*-value
Main	All population	Before overlap weighting	1.21	0.80	–	1.81	0.36
		After overlap weighting	1.13	0.74	–	1.73	0.57
Stratified	1–5 years	Before overlap weighting	0.82	0.40	–	1.67	0.58
		After overlap weighting	0.90	0.40	–	2.01	0.79
	6–11 years	Before overlap weighting	1.24	0.69	–	2.24	0.47
		After overlap weighting	1.18	0.65	–	2.16	0.59
	12–15 years	Before overlap weighting	1.98	0.63	–	6.25	0.24
		After overlap weighting	1.87	0.58	–	6.07	0.30
	Female	Before overlap weighting	1.43	0.69	–	2.98	0.34
		After overlap weighting	1.16	0.54	–	2.51	0.70
	Male	Before overlap weighting	1.12	0.69	–	1.83	0.64
		After overlap weighting	1.11	0.67	–	1.86	0.68
Sensitivity analysis 1	Generalized estimating equation using PS matched population		0.95	0.48	–	1.88	0.89
Sensitivity analysis 2	All	Instrumental variable method	1.18	0.74	–	1.89	0.49

*PS* propensity score

Age-stratified analysis showed that, when compared with general anesthesia, a multivariable adjusted relative risk for the use of intravenous sedation only was 0.90 (95% CI: 0.40–2.01), 1.18 (95% CI: 0.65–2.16), and 1.87 (95% CI: 0.58–6.07) in the three age categories (1–5, 6–11, 12–15 years, respectively). Sex-stratified analysis showed that female children had an adjusted relative risk of 1.16 (95% CI: 0.54–2.51), while male children had an adjusted relative risk of 1.11 (95% CI: 0.67–1.86) (Table 4).

**Fig. 3:**
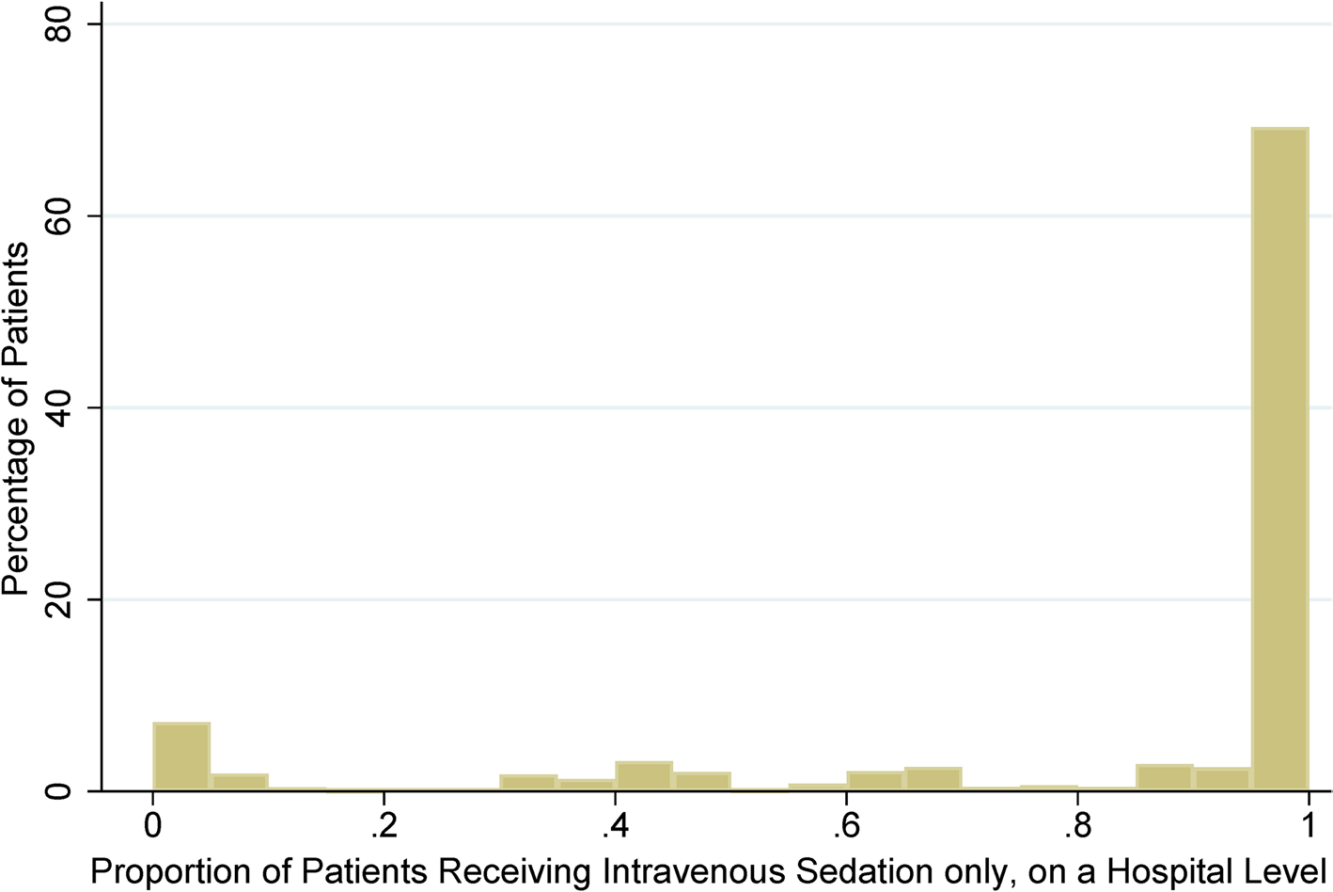
Proportion of hospitals choosing intravenous sedation only for patients admitted for kidney biopsy

In the sensitivity analysis using propensity score matching and consideration of cluster effects using generalized estimating equations, the adjusted relative risk of using intravenous sedation only was 0.95 (95% CI: 0.48–1.88). The proportions of clinicians choosing general anesthesia for patients admitted for kidney biopsy on a hospital level are shown in Fig. 3. More than half of the patients were admitted to hospitals using exclusively intravenous sedation only. The instrumental variable analysis using preference for intravenous sedation only as an instrumental variable revealed that the use of intravenous sedation only was associated with an adjusted relative risk of 1.18 (95% CI: 0.74–1.89).

## Discussion

In the present observational study using a nationwide inpatient database in Japan, the risk of bleeding complications was comparable between the intravenous sedation and general anesthesia groups. Previous studies have examined the association between anesthesia and outcomes following invasive procedures other than kidney biopsy, including otolaryngology procedures [5] and lumbar puncture or bone marrow aspiration [23]. However, these studies were limited by single-center designs and small sample sizes (N = 60–100). Our study overcame the limitations of previous studies. These insights confirm the comparable safety of general anesthesia and intravenous sedation for kidney biopsies in the pediatric population.

The total complication rate in this study (2.7%) was lower than that reported in a meta-analysis where the outcome was set as developing hematoma following biopsy (11–18%) [4]. This is probably because hematoma or hemorrhage development, in our study, was defined by the corresponding disease names occurring after hospitalization; the sensitivity of diagnostic names was shown to be generally low in previous studies [9]. On the other hand, the proportion of transfusions or invasive hemostasis (0.05%) was also lower than that in the meta-analysis (0.7%). Because the recorded procedures in the Diagnosis Procedure Combination database had a high specificity [9], our study suggests that pediatric kidney biopsy in Japan may involve a lower rate of complication occurrence than is observed in other countries.

Our results regarding incidence of severe complications (0.05%) are supported by the questionnaire survey performed between 2015 and 2017 by the Japanese Society of Nephrology, in which the proportion of severe complications as defined in this article was 0.06%. [24] Meanwhile, although incidence of non-severe complications was possibly underestimated as mentioned above, their observation may not be biased by the type of sedation. Kidney Biopsy Guidebook 2020 in Japan by the Japanese Society of Nephrology proposed clinical pathways for pediatric patients admitted for kidney biopsy and recommended ultrasound examination for bleeding both on the day and the next day of percutaneous biopsy, while it does not recommend an alternative means for biopsy and follow-up based on the type of sedation used during percutaneous biopsy. [25] This suggests that the detection of hematoma after kidney biopsy is independent of the type of sedation and thus is not biased.

The strength of our study is the use of a nationwide inpatient database, which generated a sample size comparable to that of a meta-analysis. We collected data from 328 hospitals throughout Japan, representing real-world pediatric kidney biopsy practice. Furthermore, we used statistical methods that reduced bias and strengthened comparability, including using propensity scores, overlap weighting, stratified analysis, generalized estimating equations, and the instrumental variable method. In addition, we used a nationwide database, which may be less susceptible to selection bias and, therefore, representative of clinical practice in Japan.

This study had some limitations. First, due to the nature of a retrospective observational study without randomization, treatment allocation was not determined at random. However, we dealt with measured confounders by considering overlap weighting, which enabled the absolute standardized differences to be almost zero. In addition, we dealt with unmeasured confounders using the instrumental variable method. Second, we could not adjust for serum creatinine levels, level of proteinuria, or other factors related to bleeding, such as platelet count or prothrombin time, because of the lack of data. Although we adjusted for patients with decreased kidney function using ICD-10 codes for acute kidney injury or chronic kidney failure, there might be some residual confounding factors, such as the gauge of the biopsy needle or information regarding who performed the biopsy (i.e., whether it was a radiologist or pediatrician) or whether the biopsy was successful. Finally, the population of this study may include a limited number of cases in which the ultrasound examination is not performed routinely; this could be a source of error. In Japan, ultrasound after the biopsy is recommended among pediatric patients, and it is reported that physicians in > 80% of the facilities routinely perform it [25]. Although there may be differences in screening frequency by the facility, relative risks after biopsy using intravenous sedation for bleeding complications, compared with general anesthesia, may not be affected by these differences.

## Conclusion

This retrospective cohort study using a nationwide database revealed that the risk of biopsy-related bleeding was comparable between intravenous sedation and general anesthesia during pediatric percutaneous kidney biopsy.

## Data Availability

The datasets used and/or analysed during the current study are available from the corresponding author upon reasonable request.

## Abbreviations

ICD-10: the International Classification of Diseases, 10th revision
BMI: body mass index
BMI-SDS: the BMI standard deviation score
CI: confidence interval
